# The Earth’s Great Age

**Published:** 1881-06

**Authors:** 


### THE EARTH'S GREAT AGE.

IN a recent lecture at San Francisco, Professor William Denton gave several striking illustrations of the earth's age. First, he said, we had evidence of the earth's great age in the tiny particles of soil beneath our feet. The great trees of California, with from 1,350 to 2,350 annual rings of vegetable growth, reveal the fact that these monarchs of the vegetable world were saplings when Nebuchadnezzar was born. The great fallen Monarch of the Forest has been estimated to have been four thousand years old, and grew from seed propagated by older parent trees, and these in turn from grandparents, whose crumbled dust forms a rich vegetable mould to nourish their younger progeny. How many such generations occurred no one can tell.

But older than all these are the glacial beds. When these ploughed their way over the surface of North America and Scandinavia they planed out mighty beds and ground and polished the uneven surface of a former age. In this remote age, the coast of New England was like that of Greenland at the present day. Few geologists will place the glacial period at less than 100,000 years. But we could go back still further. In the tertiary strata of California has been found what are called the earliest human remains ever discovered. These existed many, many thousand years ago, when one-half of New Jersey, one-third of Virginia, all of Florida, part of Texas and Great Britain were under water. The Mediterranean Sea was then double its present size, and the Gulf of Mexico extended to Ohio. A large part of California was under the bed of the Pacific Ocean, and waters then extended back to the foot-hills of the Sierra Nevada Mountains.

But older than this period and formation was the underlying stratification of chalk; still older was the Triassic, and older yet, the new red sandstone. Older yet was the carboniferous formation. Then further back was the old red sandstone, such as comes to the surface in parts of Scotland. Again, still lower, the older Silurian, then the older Laurentin, seen at the surface in Western Canada, and older yet than all these the granite or great underlying rock, the parent that thrusts itself up as the backbone of continents, cutting through all others, to show us on the surface what is below. What an infinity of time must have passed away in the successive formation of these rocky layers!

